# *Centella asiatica (L.)* Urb. Prevents Hypertension and Protects the Heart in Chronic Nitric Oxide Deficiency Rat Model

**DOI:** 10.3389/fphar.2021.742562

**Published:** 2021-12-03

**Authors:** Mohd Khairulanwar Bunaim, Yusof Kamisah, Mohd Noor Mohd Mustazil, Japar Sidik Fadhlullah Zuhair, Abdul Hamid Juliana, Norliza Muhammad

**Affiliations:** ^1^ Department of Pharmacology, Faculty of Medicine, Universiti Kebangsaan Malaysia, Kuala Lumpur, Malaysia; ^2^ Department of Biomedical Sciences and Therapeutics, Faculty of Medicine and Health Sciences, Universiti Malaysia Sabah, Kota Kinabalu, Malaysia

**Keywords:** *Centella asiatica*, cardiac damage, hypertension, nitro-l-arginine methyl ester, nitric oxide

## Abstract

**Background:** Hypertension is a major risk factor for cardiovascular disease (CVD), which is the number one cause of global mortality. The potential use of natural products to alleviate high blood pressure has been demonstrated to exert a cardioprotective effect. *Centella asiatica (L.)* Urb. belongs to the plant family Apiaceae (Umbelliferae). It contains a high amount of triterpenoid and flavonoid that have antioxidant properties and are involved in the renin-angiotensin-aldosterone system which is an important hormonal system for blood pressure regulation.

**Objective:** This study aimed to investigate the effects of *C. asiatica* ethanolic extract on blood pressure and heart in a hypertensive rat model, which was induced using oral N(G)-nitro-l-arginine methyl ester (l-NAME).

**Methods:** Male Sprague-Dawley rats were divided into five groups and were given different treatments for 8 weeks. Group 1 only received deionized water. Groups 2, 4, and 5 were given l-NAME (40 mg/kg, orally). Groups 4 and 5 concurrently received *C. asiatica* extract (500 mg/kg, orally) and captopril (5 mg/kg, orally), respectively. Group 3 only received *C. asiatica* extract (500 mg/kg body weight, orally). Systolic blood pressure (SBP) was measured at weeks 0, 4, and 8, while serum nitric oxide (NO) was measured at weeks 0 and 8. At necropsy, cardiac and aortic malondialdehyde (MDA) contents, cardiac angiotensin-converting enzyme (ACE) activity, and serum level of brain natriuretic peptide (BNP) were measured.

**Results:** After 8 weeks, the administrations of *C. asiatica* extract and captopril showed significant (*p* < 0.05) effects on preventing the elevation of SBP, reducing the serum nitric oxide level, as well as increasing the cardiac and aortic MDA content, cardiac ACE activity, and serum brain natriuretic peptide level.

**Conclusion:** *C. asiatica* extract can prevent the development of hypertension and cardiac damage induced by l-NAME, and these effects were comparable to captopril.

## Introduction

Cardiovascular disease (CVD) remains the number one cause of global mortality with hypertension contributes to the major risk factor (World Health Organization, 2017). Hypertension promotes mechanical stress that induces cardiomyocyte hypertrophy, apoptosis, and remodeling. Subsequently, the heart can change its size, shape, structure, and function with a consequent cardiac dysfunction (Nadruz, 2015). There is growing evidence showing that the development of hypertension and cardiac damage is associated with elevation of oxidative stress status, suppression of nitric oxide (NO) synthesis in the vasculature, and overactivation of the renin-angiotensin-aldosterone system (RAAS) (Schulz et al., 2011; Münzel et al., 2017; Bakogiannis et al., 2019; Wunpathe et al., 2020).

Elevation of oxidative stress is largely attributable to excessive production of reactive oxygen species (ROS) such as superoxide, hydrogen peroxide, and hydroxyl radicals, which are toxic byproducts of human aerobic metabolism (Sedeek et al., 2009). Importantly, an increase in oxidative stress profile induces endothelial dysfunction, inflammatory processes, and vascular smooth muscle tone (Touyz and Briones, 2011). Meanwhile, NO that is produced in endothelial tissues, activates soluble guanylate cyclase to produce 3′,5′-cyclase guanosine monophosphate (cGMP), which has an important role in vasodilatory processes, inhibition of platelet adhesion, and smooth muscle proliferation (Zhou et al., 2004). This vasodilatory action reduces vascular resistance and blood circulatory pressure, which eventually contributes to a lower risk of hypertension. As expected, vascular NO deficiency is a major finding in dysfunctional endothelium and arterial hypertension (Lüscher and Vanhoutte, 1986; Panza et al., 1990). RAAS is an important neurohormonal system that involves the regulation of blood pressure and tissue perfusion. Angiotensin-converting enzyme (ACE) is one of the RAAS components and it converts angiotensin I (AT-I) into angiotensin II (AT-II). If this hormonal system is overactivated, AT-II will be released excessively and chronically, leading to vasoconstriction, aldosterone secretion, activation of the sympathetic nervous system, anti-natriuretic mechanism, and hypertension. Besides that, AT-II can be produced locally in the heart, primarily induced by an increase in cardiac wall stress. The binding of AT-II to angiotensin II type I receptor (AT1R) stimulates the formation of heart collagen by fibroblast cells, which subsequently increases the risk for cardiac hypertrophy, fibrosis, and cardiomyocyte apoptosis (Singh et al., 2008; Dai et al., 2011; Jia et al., 2012). For instance, AT-II infusion for 2 weeks induced cardiac hypertrophy and elevated oxidative stress markers in rat cardiac tissue (Kakishita et al., 2003).

Despite a variety of anti-hypertensive agents such as ACE inhibitors, angiotensin II receptor blockers (ARB), β-adrenergic receptor blocker, and calcium channel antagonists are being used in the treatment of hypertension and cardiac hypertrophy, the progression of myocardial injury and subsequent cardiac dysfunction remains a major problem in chronic hypertensive subjects (Eirin et al., 2014). There has been considerable interest in the potential use of natural products to alleviate blood pressure, which has been demonstrated to exert a cardioprotective effect. One of them is *Centella asiatica (L.)* Urb., which is also known as Indian pennywort, abundantly found in tropical countries including China, India, and the South-East Asian countries (Brinkhaus et al., 2000). Apart from fever and wound healing, this medicinal plant was used traditionally to treat stomach upset, leprosy, bladder inactivity, and urinary tract infection (Brinkhaus et al., 2000). Triterpenoid compounds such as asiatic acid, madecassic acid, asiaticoside, and madecassoside are the major biologically active compounds in *C. asiatica* (James and Dubery, 2009). Other compounds isolated from this plant are flavonoids, brahmoside, brahminoside, glycosides isothankuniside, and thankuniside (Azerad, 2016). In modern medicine, *C. asiatica* has been reported to have high antioxidant property due to its abundant triterpenoid and flavonoid contents (Pittella et al., 2009). The herb and its bioactive compounds may have a role in therapeutic application in diseases associated with oxidative stress, such as hypertension and cardiac failure. Studies using triterpenoid- and flavonoid-rich chloroform fractions of *C. asiatica* in a hypertensive rat model showed the effectiveness of the extract in reducing the acute rise in blood pressure with a single dose (Harwoko and Nugroho, 2014; Nansy et al., 2015). In addition, asiatic acid, one of the main triterpenoid compounds in *C. asiatica*, was reported to prevent RAAS activation in metabolic syndrome rats (Maneesai et al., 2016a). Furthermore, flavonoid-rich fruits such as apple and kiwi inhibited ACE activity in some *in vitro* studies (24, 25), suggesting similar potential cardiovascular benefits offered by *C. asiatica*.

Although previous *in vivo* studies have reported the anti-hypertensive effect of *C. asiatica* (Harwoko and Nugroho, 2014; Nansy et al., 2015), those studies were carried out either in a single dosage of *C. asiatica* extract or by bioactive compounds isolated from the plant. To date, there has been no studies that investigate the effects of *C. asiatica* extract on chronic hypertension and cardiac damage. Therefore, to bridge the research gap, this study was conducted to explore the blood pressure-lowering and cardioprotective effects of *C. asiatica* in chronic hypertensive rats. N(G)-nitro-l-arginine methyl (l-NAME) was administered to induce systemic hypertension and cardiac damage in rats.

## Materials and Methods

### Drugs, Chemicals and Kits

l-NAME and other chemicals were obtained from Sigma-Aldrich (St. Louis, MO, United States) unless stated otherwise. Captopril tablet was purchased from Y.S.P Industries (Selangor, Malaysia). The kits for cardiac ACE activity and serum brain natriuretic peptide (BNP) level measurements were purchased from Elabscience (Wuhan, China).

### Plant Material and Extraction

The fresh leaves of *C. asiatica* were collected in February 2020 from Kepala Batas, Pulau Pinang, Malaysia. The specimen was identified by a botanist in Universiti Kebangsaan Malaysia Herbarium and a voucher specimen of the plant (UKMB 40434) was deposited at that institute. The cold extraction method was employed using the maceration technique. A 1 kg of the dried leaves was soaked in 4 L of 80% ethanol and agitated on a shaker at 16 rpm for 24 h. The extracts were filtered and the residual sample was soaked again two more times using fresh ethanol, making a total of 12 L. The combined extracts were concentrated and freed of solvent using a rotary evaporator, then dried in a freeze-dryer. The yield percentage of the ethanolic extract was 16.6%.

### Animals

Two-month-old male Sprague-Dawley rats (200–250 g) were obtained from Laboratory Animal Resources Unit, Universiti Kebangsaan Malaysia. They were maintained at room temperature (27 ± 2°C) with natural light at the Laboratory Animal Center, Department of Pharmacology, Universiti Kebangsaan Malaysia. All procedures were complied with the standards for the care and use of experimental animals and were approved by the Animal Ethics of Universiti Kebangsaan Malaysia (FAR/PP/2019/NORLIZA/30-OCT./1048-OCT.2019-AUG.-2020). Rats were fed with a commercial diet (Gold Coin Feed-mills (M) Sdn Bhd, Selangor, Malaysia) and tap water *ad libitum*.

### Experimental Protocol

Thirty rats were randomly divided into five groups (*n* = six rats/group). At a baseline, rat blood was taken via cardiac puncture after being given ketamine (0.1 ml/100 g body weight) and xylazine (0.01 ml/100 g body weight) via intraperitoneal injection as anesthesia. Groups 2, 4, and 5 were given l-NAME (40 mg/kg body weight, orally) for 8 weeks. Groups 4 and 5 concurrently received *C. asiatica* extract (500 mg/kg body weight, orally) and captopril (5 mg/kg, orally), respectively. Groups 1 and 3 only received deionized water and *C. asiatica* extract (500 mg/kg, orally body weight) respectively. The dose of the extract was selected based on a pilot study (Supplementary Material). All treatments were given every day for 8 weeks via oral gavage. Body weight was measured at the baseline and after the study ended. After 8 weeks of study, blood was taken via cardiac puncture, and all animals were sacrificed. Heart and aortic tissues were harvested after completing the study for biochemical analysis.

### Indirect Measurement of Blood Pressure in Conscious Rats

Animal systolic blood pressure (SBP) was measured at baseline, fourth week and eighth week of study using non-invasive tail-cuff plethysmography (Kent Scientific Cooperation, Torrington, US). In brief, conscious rats were placed in a restrainer and allowed to be calm before SBP measurement. The rat tail was placed inside the tail-cuff, and the cuff was automatically inflated and released. For each rat, five stable SBP readings were chosen and averaged from each cycle.

### Biochemical Analysis

Serum nitric oxide (NO) level was measured using Griess reagent against sodium nitrite standard curve. It was expressed as the percentage difference between weeks 0 and 8. Cardiac and aortic malondialdehyde (MDA) contents were measured as thiobarbituric acid reactive substance (TBARS) according to the method of Ledwozyw et al. (1986). Malondialdehyde tetraethyl acetal was used as the standard and the TBARS content was expressed as MDA per protein (μmol/mg). The protein content was determined based on the method described by Bradford (1976). Cardiac ACE activity and serum BNP level were determined using a commercial kit (Elabscience, Wuhan, China) based on the sandwich enzyme immunoassay principle.

### Statistical Analysis

All results were expressed as mean ± standard error of the mean (SEM). The data were analyzed for normality test using the Shapiro-Wilk test. Analysis of variance (ANOVA) with Bonferroni posthoc test was used for the SBP parameter. The differences between the groups for body weight, NO, MDA, BNP, and ACE parameters were compared using one-way ANOVA with Tukey’s Honestly Significant Differences posthoc test. Statistical significance was defined as *p* < 0.05. Statistical analyses were conducted using the Statistical Product for Social Science 23 software (SPSS Inc., Chicago, IL).

## Results

### Body Weight

**FIGURE 1:**
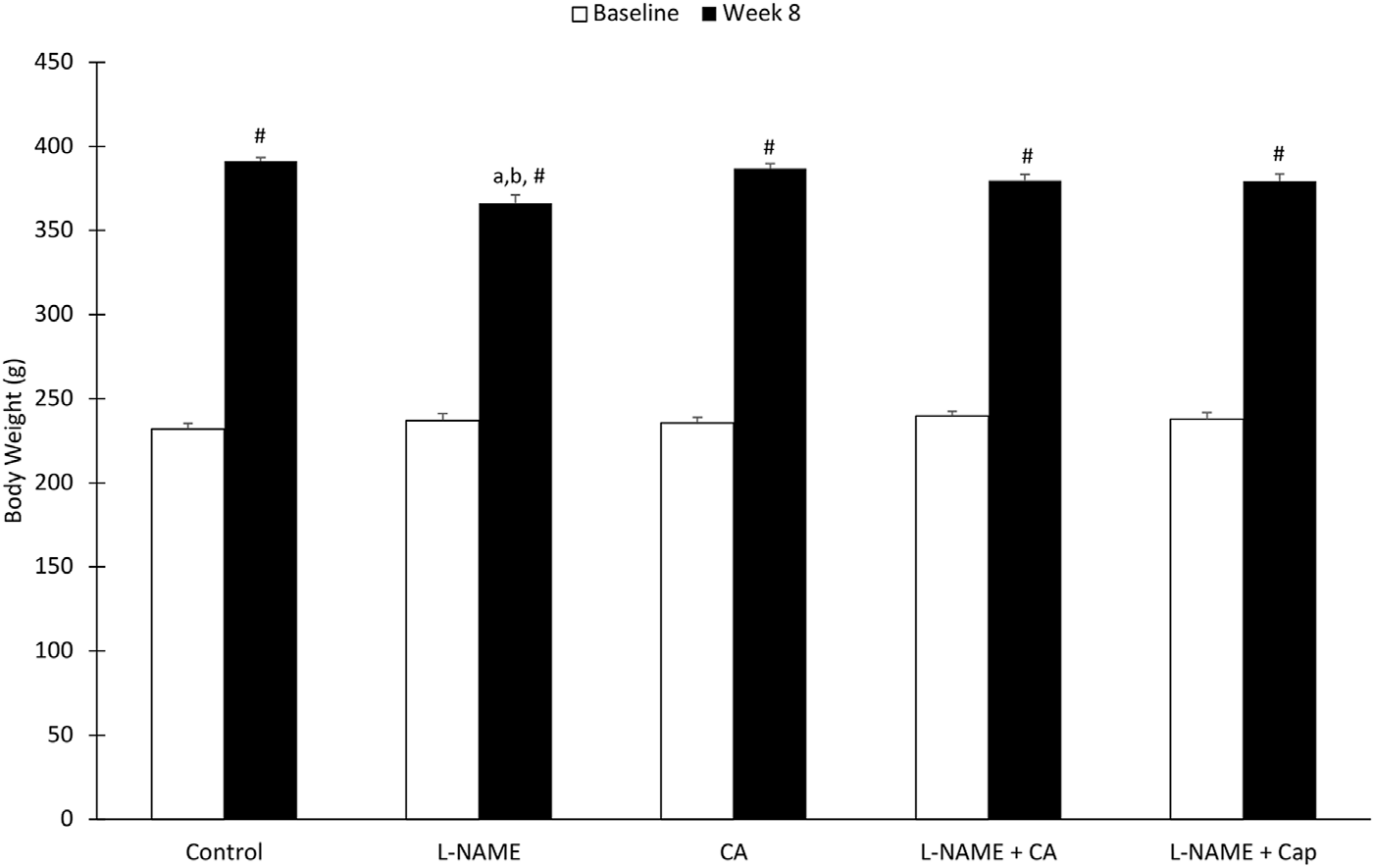
Rat body weight before and after administration of l-NAME (40 mg/kg orally) and *C. asiatica* extract (500 mg/kg orally) or captopril (5 mg/kg orally) during the 8-weeks of study. The values represent mean ± SEM (*n* = 6). ^#^versus baseline (*p* < 0.05), ^a^versus control (*p* < 0.05), ^b^versus *C. asiatica* (*p* < 0.05).

There was no significant difference (*p* > 0.05) in body weight among the rat groups at the baseline (Figure 1). After 8 weeks of treatment, there was a significant increase (*p* < 0.05) in body weight seen in all groups compared to the baseline. However, group 2 (366.17 ± 5.04 g) demonstrated a significantly lower (*p* < 0.05) body weight when compared to the control group (391.17 ± 2.09 g).

### Systolic Blood Pressure

**FIGURE 2:**
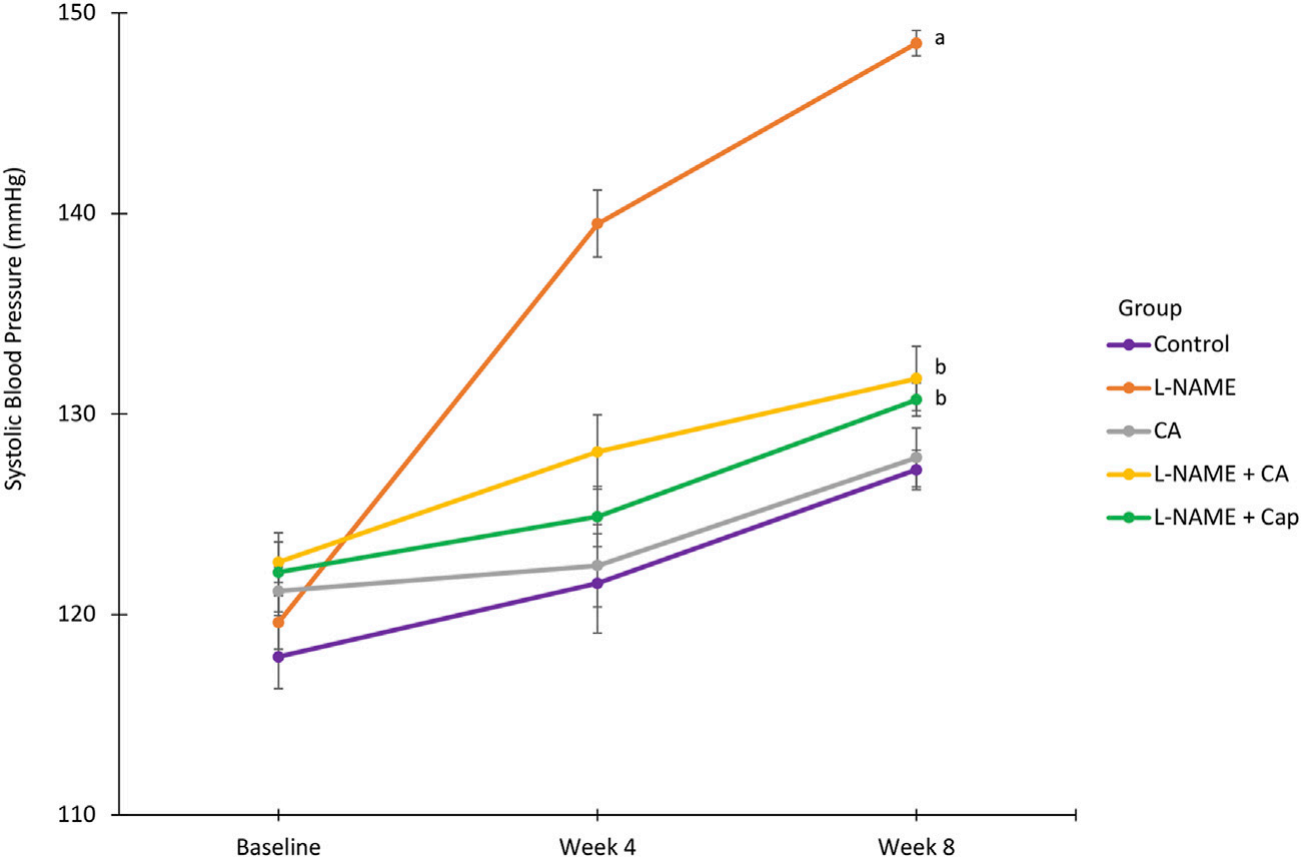
Systolic blood pressure in rats treated with concurrent l-NAME (40 mg/kg orally) and *C. asiatica* (500 mg/kg orally) or captopril (5 mg/kg orally). Line graph represent mean ± SEM (*n* = 6). ^a^versus control (*p* < 0.05), ^b^versus l-NAME (*p* < 0.05).

SBP was significantly elevated (*p* < 0.05) in group 2 (148.50 ± 0.64 mmHg) with chronic l-NAME exposure when compared with the control group (group 1) after 8 weeks (127.22 ± 0.99 mmHg) (Figure 2). The increase in SBP induced by l-NAME was attenuated in groups 4 and 5 given *C. asiatica* (131.78 ± 1.60 mmHg) and captopril (130.72 ± 0.83 mmHg), respectively. No significant difference (*p* > 0.05) was noted between these two groups. *C. asiatica* had no effect (*p* > 0.05) on SBP in *C. asiatica* control rats of group 3 (127.83 ± 1.46 mmHg).

### Serum Nitric Oxide Level

**FIGURE 3:**
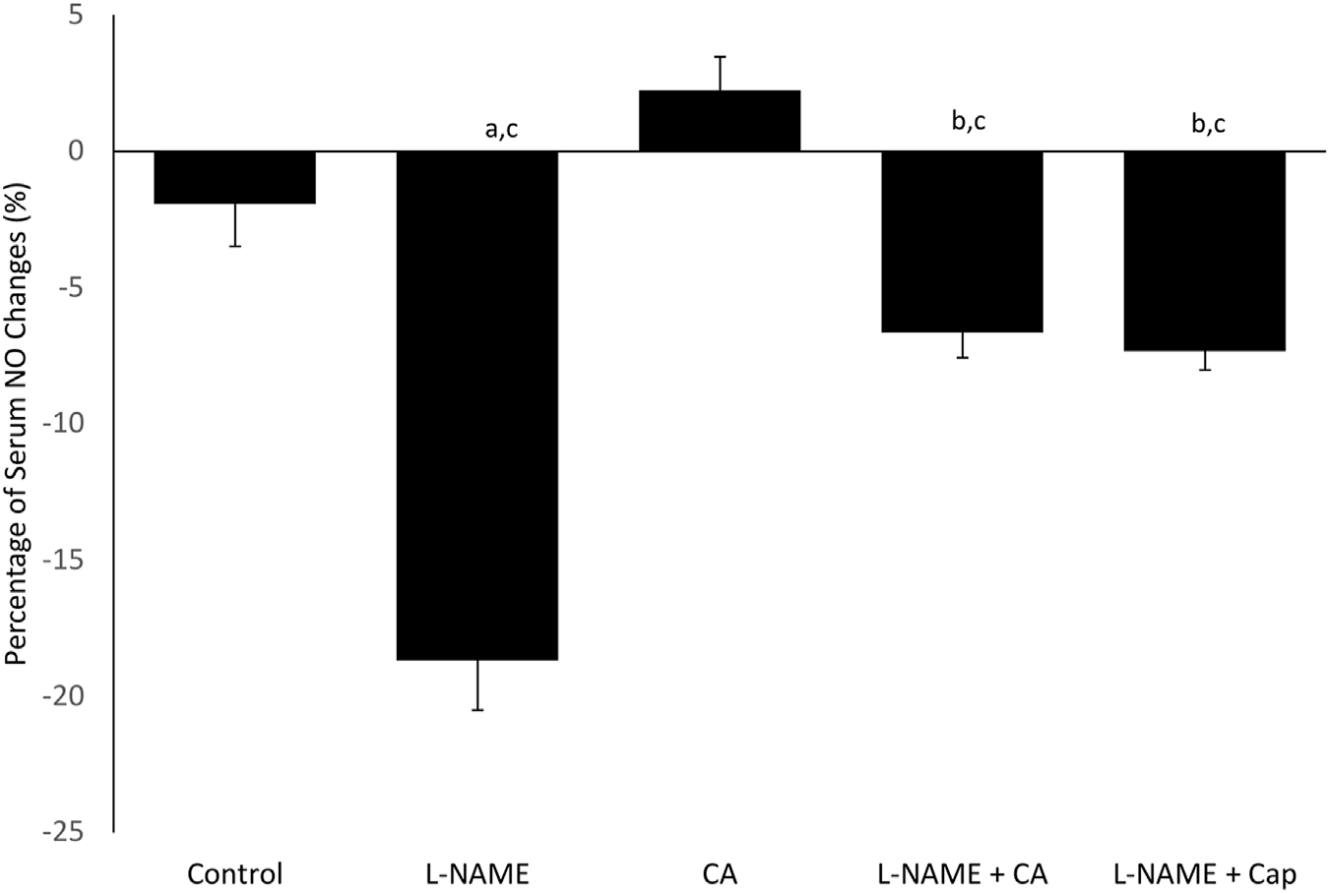
Percentage of plasma nitric oxide (NO) change in rats given *C. asiatica* extract (500 mg/kg orally) and captopril (5 mg/kg orally) together with l-NAME (40 mg/kg orally) for 8 weeks. Bars represent mean ± SEM (*n* = 6). ^a^versus control (*p* < 0.05), ^b^versus l-NAME (*p* < 0.05), ^c^versus *C. asiatica* (*p* < 0.05).

A significant (*p* < 0.05) reduction in serum NO was seen in the group 2 (−18.68 ± 1.81% mmHg) after 8 weeks compared to the control of group 1 (−1.94 ± 1.54% mmHg) (Figure 3). Concurrent treatment of l-NAME administered in groups 4 and 5 with *C. asiatica* (−6.65 ± 0.92% mmHg) and captopril (−7.33 ± 0.69% mmHg), respectively had significantly (*p* < 0.05) prevented the NO reduction induced by l-NAME.

### Cardiac Angiotensin-Converting Enzyme Activity

**FIGURE 4:**
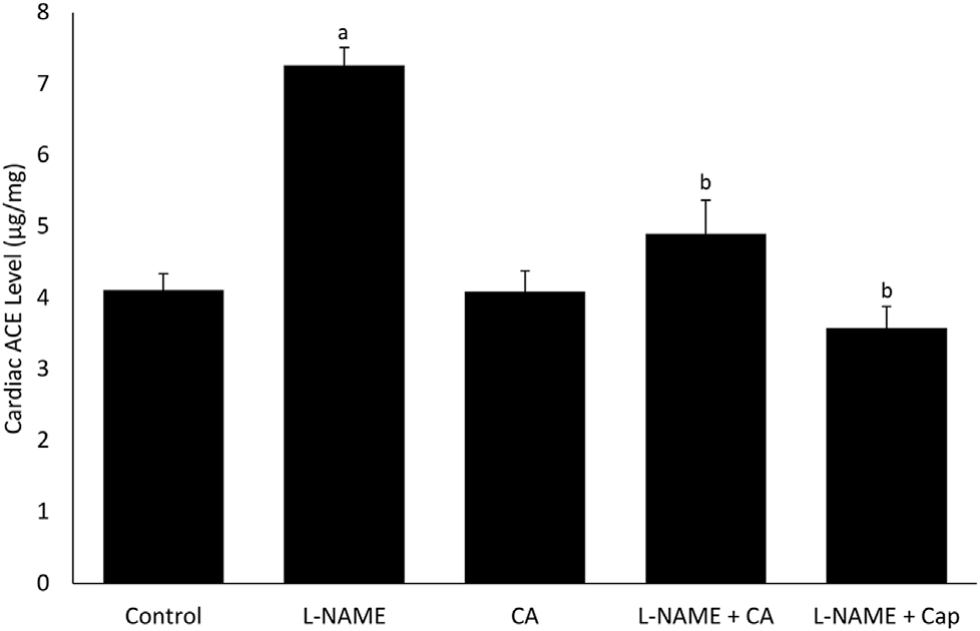
Cardiac angiotensin-converting enzyme (ACE) activity in rats treated with *C. asiatica* extract (500 mg/kg orally) or captopril (5 mg/kg orally) together with l-NAME (40 mg/kg orally) for 8 weeks. Bars represent mean ± SEM (*n* = 6). ^a^versus control (*p* < 0.05), ^b^versus l-NAME (*p* < 0.05).

The ACE activity in the heart was significantly (*p* < 0.05) elevated in the group 2 (7.26 ± 0.25 μg/mg) as compared to the control of group 1 (4.11 ± 0.23 μg/mg). The elevation was inhibited by the concurrent treatment of *C. asiatica* (4.90 ± 0.47 μg/mg) and captopril (3.58 ± 0.30 μg/mg) in groups 4 and 5, respectively (Figure 4). No significant (*p* > 0.05) difference in cardiac ACE activity was observed among groups 1, 2, and 5.

### Cardiac and Aortic Thiobarbituric Acid Reactive Substance Content

**FIGURE 5:**
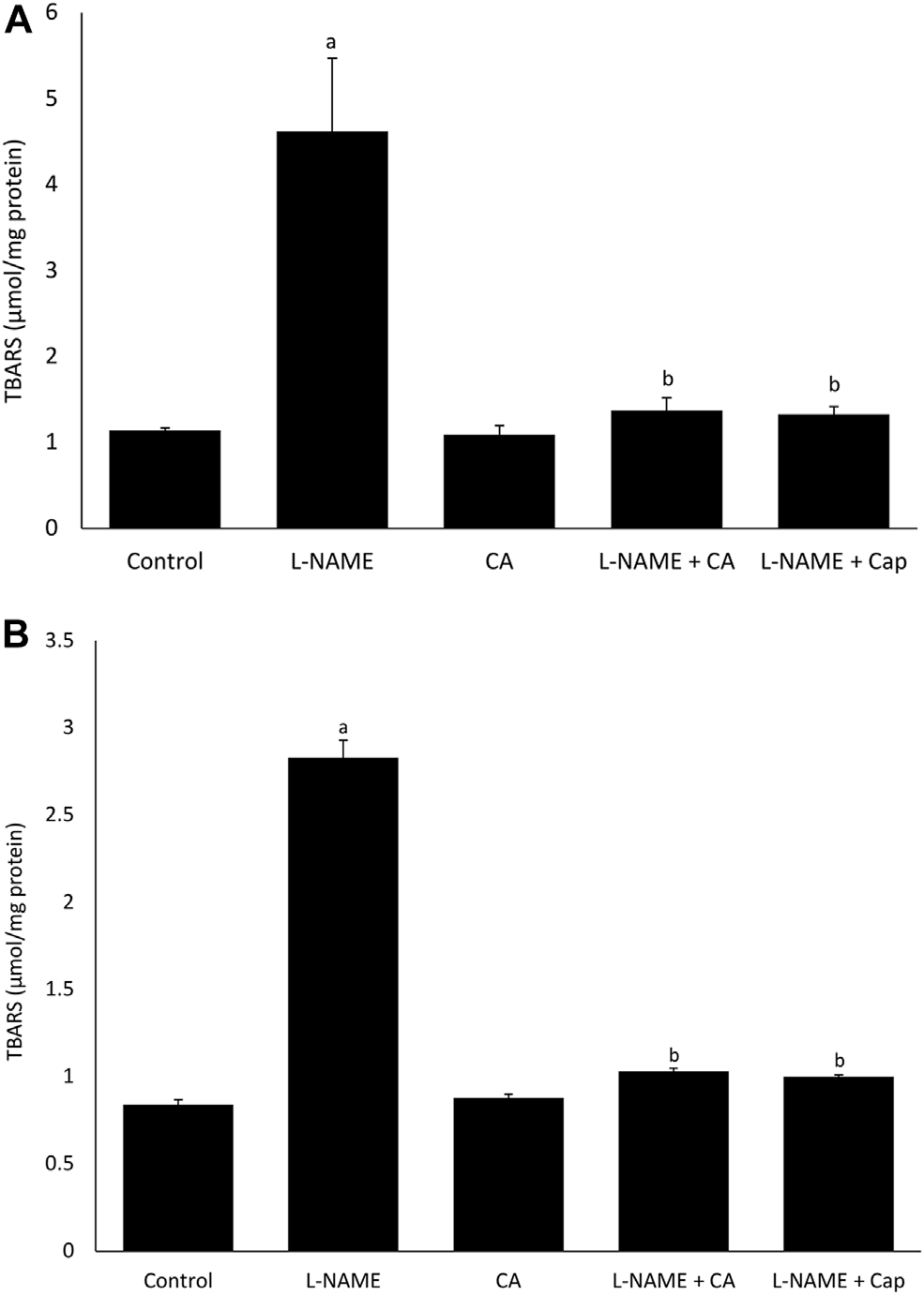
The effects of *C. asiatica* (500 mg/kg orally) and captopril (5 mg/kg orally) on **(A)** aortic and **(B)** cardiac TBARS content in l-NAME-administered rats (40 mg/kg orally) after 8 weeks. Bars represent mean ± SEM (*n* = 6). ^a^versus control (*p* < 0.05), ^b^versus l-NAME (*p* < 0.05).

l-NAME administration in group 2 for 8 weeks significantly (*p* < 0.05) increased cardiac and aortic TBARS content when compared to the control group (group 1) (Figures 5A,B). Treatments with *C. asiatica* and captopril in groups 4 and 5, respectively had similarly prevented the elevation of TBARS content induced by l-NAME in both heart and aorta.

### Serum Brain Natriuretic Peptide Level

**FIGURE 6:**
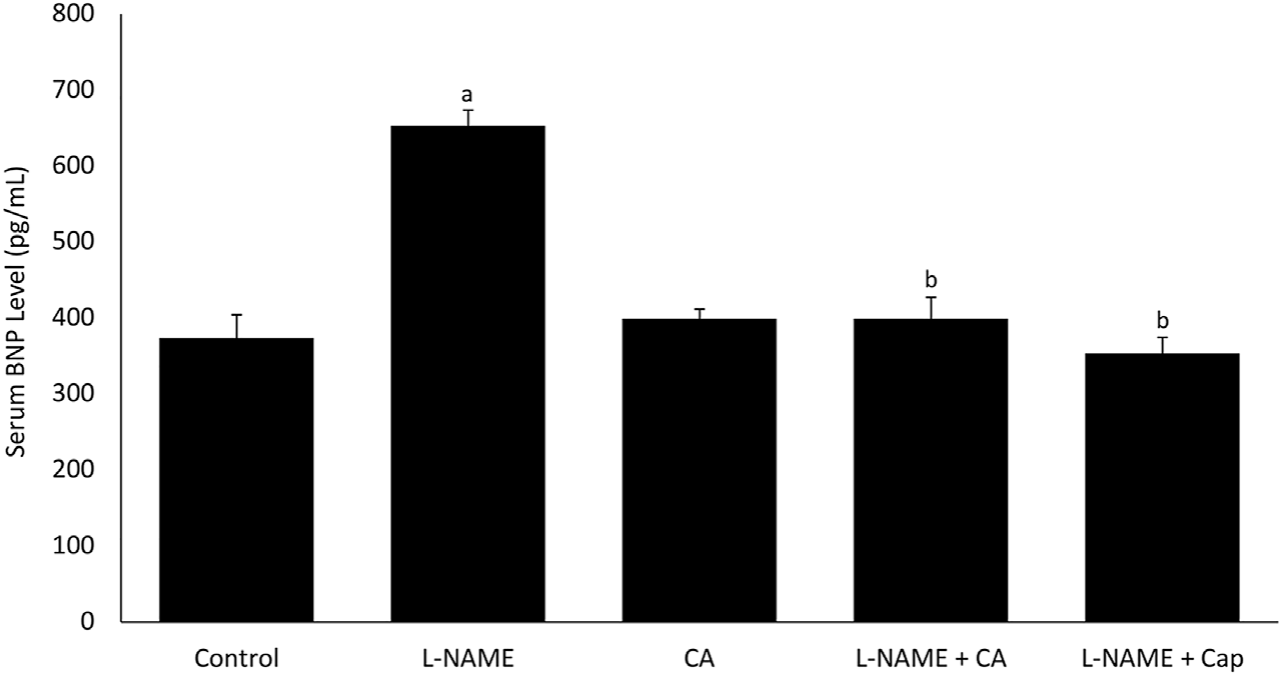
Serum B-natriuretic peptide (BNP) level in rats co-treated with l-NAME (40 mg/kg orally) and *C. asiatica* extract (500 mg/kg orally) or captopril (5 mg/kg orally) for 8 weeks. Bars represent mean ± SEM (*n* = 6). ^a^versus control (*p* < 0.05), ^b^versus l-NAME (*p* < 0.05).

Serum BNP level was significantly (*p* < 0.05) higher in group 2 (652.42 ± 20.79 pg/ml) receiving l-NAME for 8 weeks than the control group 1 (373.56 ± 30.59 pg/ml) (Figure 6). Treatments with *C. asiatica* (398.88 ± 28.17 pg/ml) and captopril (353.27 ± 21.18 pg/ml) in groups 4 and 5, respectively had similarly prevented the rise in the serum BNP level.

## Discussions

The present study exhibited the effects of ethanolic extract of *Centella asiatica (L.)* Urb. on the cardiovascular system in l-NAME-induced hypertensive rats as summarized in Table 1. Ethanol extraction was chosen because it was proven to produce a high yield of flavonoid (Andarwulan et al., 2010; Nansy et al., 2015) and triterpenoids such as asiatic acid, asiaticoside, madecassic acid, and madecacosside (Hashim et al., 2011). Nansy et al. (2015) and Harwoko and Nugroho (2014) showed that triterpenoid and flavonoid-rich fractions isolated from ethanolic extract of *C. asiatica* and administered at bolus dose, respectively, reduced blood pressure in phenylephrine-induced hypertensive rats. Asiatic acid, another bioactive compound in *C. asiatica*, had been shown to exert significant anti-hypertensive effect in numerous types of hypertensive rat model (Bunbupha et al., 2014; Maneesai et al., 2016a; Maneesai et al., 2017). The ethanolic extract was also reported to possess cardioprotective benefit in myocardial infarction (Pragada et al., 2004) in addition to its high antioxidant activities (Muchtaromah et al., 2016). Nevertheless, we could not find any literature on the cardioprotective ability of *C. asiatica* extracted using other methods.

**TABLE 1:** Summary of result from measured parameters. The values represent mean ± SEM (*n* = 6).

	Control	l-NAME	*C. asiatica*	l-NAME + *C. asiatica*	l-NAME + captopril
Baseline Body Weight (g)	232.00 ± 3.34	237.00 ± 4.16	235.50 ± 3.36	239.83 ± 2.66	237.67 ± 4.12
Final Body Weight (g)	391.17 ± 2.09	366.17 ± 5.04^a^ ^,^ ^b^	386.67 ± 2.95	379.67 ± 3.65	379.33 ± 4.39
Baseline Systolic Blood Pressure (mmHg)	117.89 ± 1.58	119.61 ± 1.33	121.17 ± 1.22	122.61 ± 1.01	121.11 ± 1.97
Final Systolic Blood Pressure (mmHg)	127.22 ± 0.99	148.50 ± 0.64^a^	127.83 ± 1.46	131.78 ± 1.60^c^	130.72 ± 0.83^c^
Serum NO Change (%)	-1.94 ± 1.54	-18.68 ± 1.81^a^ ^,^ ^b^	2.24 ± 1.23	-6.65 ± 0.92^b^ ^,^ ^c^	-7.33 ± 0.69^b^ ^,^ ^c^
Cardiac ACE (μg/mg)	4.11 ± 0.23	7.26 ± 0.25^a^	4.09 ± 0.29	4.90 ± 0.47^c^	3.58 ± 0.30^c^
Aortic TBARS (μmol/mg protein)	1.14 ± 0.03	4.62 ± 0.85^a^	1.09 ± 0.11	1.37 ± 0.15^c^	1.33 ± 0.09^c^
Cardiac TBARS (μmol/mg protein)	0.84 ± 0.03	2.83 ± 0.10^a^	0.88 ± 0.02	1.03 ± 0.02^c^	1.00 ± 0.01^c^
Serum BNP (pg/ml)	373.56 ± 30.59	652.42 ± 20.79^a^	399.06 ± 12.41	398.88 ± 28.17^c^	353.27 ± 21.18^c^

a versus control (p < 0.05).

b versus L-NAME (p < 0.05).

c versus C. asiatica (p < 0.05).

In the current study, we used l-NAME to induce chronic hypertension in our rats. It appeared that this compound had a detrimental effect on the rat’s growth as evidence by the reduction in body weight. Chronic l-NAME administration was shown to attenuate weight gain in normal and high-fat diet-fed rats, possibly due to decreased food intake in the l-NAME group (Tsuchiya et al., 2007). Nonetheless, concomitant treatment with *C. asiatica* reversed the effect in the present study. This herbal extract was capable of overcoming this noxious effect on weight gain, conceivably due to its high flavonoid content, which contributed to lower energy consumption and a more efficient digestive system (Ke et al., 2015). The safety of *C. asiatica* consumption was proven in an acute toxicity testing, in which *C. asiatica* extract at the maximum dose of 2000 mg/kg body weight showed no toxic signs and mortality in rats. In a subacute toxicity testing, there was no significant alteration in body weight, general health, and food intake in rats after receiving *C. asiatica* extract ranged from 10 to 1,000 mg/kg/day for 90 days in comparison to the control group (Kumari et al., 2016).

**FIGURE 7:**
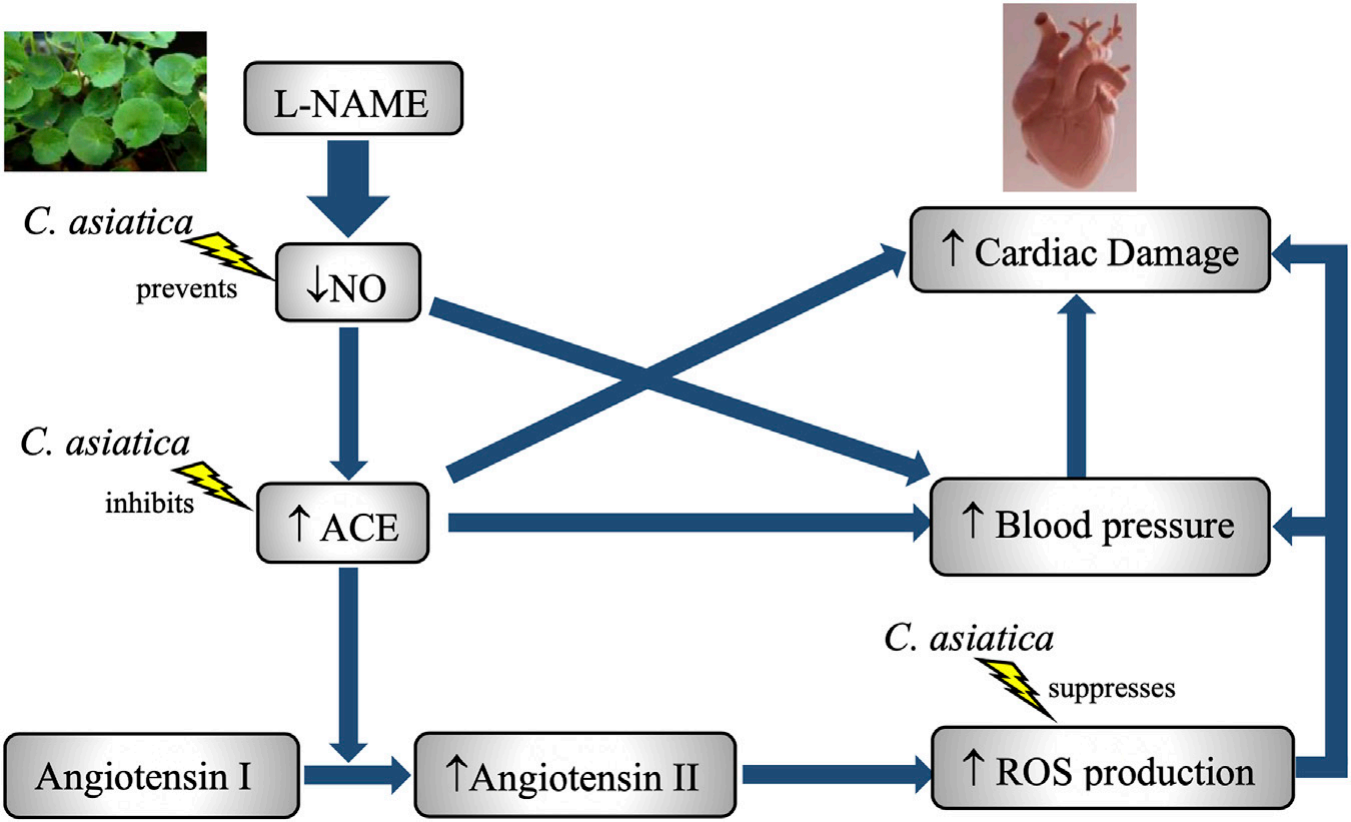
A schematic diagram on the role of NO deficiency in l-NAME-induced hypertension and cardiotoxicity in rats. Treatment with *C. asiatica* extract exhibited anti-hypertensive and cardioprotective effects via the enhancement of NO bioavailability, the suppression of RAAS and amelioration of oxidative stress status in l-NAME-treated group.

The paradigm of using l-NAME to establish experimental hypertension has become a widely accepted method for testing anti-hypertensive drugs. Our observation that chronic eNOS inhibition is responsible for the development of hypertension, which is also supported by other studies (Sung et al., 2013; Bunbupha et al., 2014). l-NAME is a potent inhibitor of eNOS that depreciates NO synthesis in cells. In blood vessels, it diminishes vascular relaxation due to a reduction in the availability of NO, a potent vasodilator. As expected, group 2 administered with l-NAME alone demonstrated a greater reduction in NO at the end of the study. This led to an increase in blood pressure, as similarly reported by Aluko et al. (2019) and Berkban et al. (2015). However, concomitant treatment of *C. asiatica* for 8 weeks managed to restore the serum NO level and eventually prevented the elevation of blood pressure, despite the chronic l-NAME exposure (Figure 7). NO promotes vasodilation via induction of soluble guanylate cyclase and increases cyclic guanosine monophosphate (cGMP) in smooth muscle cells (Capettini et al., 2010; Förstermann and Sessa, 2012). Previous evidence suggested that a high content of asiatic acid in *C. asiatica* might contribute to the normalization of serum NO level and blood pressure, as reported in a range of different experimental models of hypertension including metabolic syndrome, renovascular, and l-NAME-induced hypertension (Bunbupha et al., 2014; Maneesai et al., 2016a; Maneesai et al., 2017). Asiatic acid also upregulated eNOS protein expression and ameliorated systemic vasodilation in these studies, further explaining the anti-hypertensive effects of *C. asiatica* on chronic NO deficiency induced by l-NAME.

Excessive lipid peroxidation alarms a weak antioxidant defense and subsequent oxidative damage that increases the oxidative status, resulting from overproduction of ROS (Yu, 1994). Oxidative stress is a pro-hypertensive factor that aggravates endothelial dysfunction and enhances vasoconstriction, which together contributes to increasing systemic vascular resistance that results in BP elevation (Sorriento et al., 2018). MDA is a product of the reaction between lipid bilayer of membrane plasma with ROS (Samhan-Arias et al., 2011), which can react with thiobarbituric acid to produce TBARS. In the l-NAME-induced hypertensive rat, increased TBARS level in the aorta indicated a worsening oxidative stress profile, which was observed similarly in other studies (Rajeshwari et al., 2014; Kamisah et al., 2017). Endogenous NO suppresses vascular p47^phox^ protein expression and superoxide production, which contribute to the source of vascular oxidative stress that initiates vascular inflammation and dysfunction (Harrison et al., 2010). Reduced bioavailability of NO may underlie the development of oxidative stress-related hypertension in the present study. Besides, a chronic NO inhibition promotes a higher expression of NADPH-dependent oxidase in aortic smooth muscle cells and a subsequent exacerbation in vascular superoxide (O_2_^−^) formation via excessive production of AT-II (Touyz and Schiffrin, 1999; Giani et al., 2014).

The augmented generation of ROS by l-NAME was overcome by *C. asiatica* in this study based on the low content of TBARS in aortic tissue, which was comparable to that of the control group. Our finding is in line with previous literature that demonstrates the antioxidant property of *C. asiatica* extract (Pittella et al., 2009; Kumari et al., 2016). The astounding ROS scavenging ability of this herb is also apparent in various models of organ injury (Wei et al., 2013; Pakdeechote et al., 2014). In a study by Bunbupha et al. (2014), asiatic acid was demonstrated to inhibit the overproduction of O_2_^−^ in aortic tissue and plasma MDA via downregulation of p47^phox^ expression, which subsequently restored the vascular function and improved the hemodynamic parameters. A similar finding was also reported by Maneesai et al. (2016a) showing that anti-hypertensive effects of asiatic acid in the metabolic syndrome model were contributed by its antioxidant and anti-inflammatory properties. Besides, protection against oxidative damage in cells is mainly depending on the scavenging enzymes, which break down free radicals including superoxide and hydroxyl, and subsequently improve the oxidative stress status (Robaczewska et al., 2016; Ahmadinejad et al., 2017). *C. asiatica* extract can ward off the free radical excess via enhancement of free radical scavenging enzymes including catalase, superoxide dismutase (SOD), glutathione peroxidase (GPx), and glutathione-S-transferase (GST), which attenuate the superoxide production, ameliorate the vascular oxidative stress, and reverse the free radical-induced cellular damage (Tabassum et al., 2013). NO can react with O_2_^−^ to produce peroxynitrite (ONOO^−^), thus mitigate the biological effect of O_2_^−^ (Pryor and Squadrito, 1995). Our current work showed an inverse relationship between NO and ROS level in *C. asiatica*-treated group, suggesting that this herbal extract reduced the ROS level via upregulation of NO bioavailability.

BNP is a biomarker that plays an important role in natriuresis, vasodilatation, RAAS inhibition, and the sympathetic nervous system (Weber and Hamm, 2006). It is released by injured cardiac tissue due to increased mechanical stretch, hypoxia, and tissue ischemia (D'Souza and Baxter, 2003; Kinnunen et al., 1993; Tóth et al., 1994). Its level correlates with mass increase and tissue damage in the cardiac ventricle (Conen et al., 2006). In the current study, administration of l-NAME increased the availability of BNP in plasma indicating that this compound could feasibly induce cardiac injury. Our observation is consistent with other studies which showed that there was an increase in other cardiac biomarkers such as troponin T and creatinine kinase-MB (CKMB) after 4 weeks of l-NAME administration (Adamcova et al., 2013; Aldubayan et al., 2020). These findings suggested the detrimental effects of l-NAME on cardiac tissue. More evidence was observed in our study, in which the deficient state of NO was associated with cardiac damage induced by l-NAME. Significant works had been done to show the role of NO in influencing cardiac performance. Early observation exhibited that NO enhanced Frank-Starling response and cardiac distensibility (Grocott-Mason et al., 1994). A later study by Casadei and Sears (2003) generated similar findings with intracardiac NO improved diastolic relaxation and cardiac stiffness. In addition, a downregulation of eNOS activity followed by reduced bioavailability of NO was reported to deteriorate the ventricular hypertrophy and dysfunction in chronic l-NAME exposure in rats (Kumar et al., 2014; Jin et al., 2017). In a more detailed framework, an interaction between eNOS and cardiac ACE activities was reported via a feedback regulation (Wiemer et al., 1997; Takemoto et al., 1997), which contributed to the pathophysiology of l-NAME-induced cardiac damage. Silambarasan et al. (2014) reported that increased myocardial dysfunction, cardiac hypertrophy, and fibrosis were positively correlated to the oxidative markers and ACE activity in nitric oxide inhibited rats. AT-II is produced during the early stage of heart failure via cardiac ACE action to compensate for the reduced cardiac function during the chronic deficient state of nitric oxide. However, as the disease progresses, RAAS is excessively activated. This results in increased cardiac preload and afterload, promotes cardiac hypertrophy, fibrosis, and cardiomyocyte apoptosis (Singh et al., 2008; Dai et al., 2011; Jia et al., 2012), and subsequently worsens the heart function (Jackson et al., 2000). In addition, cardiac AT-II also enhances the mitochondrial ROS generation in cardiomyocytes including the excessive production of NADPH (De Giusti et al., 2009; De Giusti et al., 2013). The elevation of oxidative stress seems to augment the progression of heart remodeling and the subsequent cardiac damage (Rababa'h et al., 2018).

Systemic administration of *C. asiatica* extract inhibited an increase in serum BNP level induced by l-NAME in our study. Possible explanations for the beneficial effects of *C. asiatica* could be the enhancement of NO bioavailability, reduction of cardiac ACE activity, and improvement of oxidative stress status. Pragada et al. (2004) reported that *C. asiatica* extract possessed a cardio-protective effect that was attributable to its high content of triterpenoid. Asiatic acid was demonstrated to prevent cardiac and aorta remodeling including left ventricular hypertrophy, myocardial fibrosis, collagen deposition, and aortic wall thickening in a chronic NO deficiency state, as reported by Bunbupha et al. (2015). Another triterpenoid compound, asiaticoside was reported to have anti-hypertensive and cardioprotective effects in pulmonary hypertension and right ventricular hypertrophy rat model via induction of PI3K/Akt/eNOS signaling pathway (Wang et al., 2018). Based on the previous literature, we speculate that the triterpenoid in *C. asiatica* upregulated eNOS activity and increased NO content in l-NAME treated rats, and these mechanisms might play a beneficial role in the amelioration of cardiac damage.

*C. asiatica* also inhibited the increase in cardiac ACE activity induced by l-NAME. Reports from the previous studies noted that asiatic acid managed to attenuate excessive RAAS activation in several models of hypertensive rats (Maneesai et al., 2016a; Maneesai et al., 2017). Some studies showed that flavonoid compounds isolated from kiwi and apple peel inhibited ACE activities *in vitro* (Balasuriya and Rupasinghe, 2012; Hettihewa et al., 2018). These findings may suggest the role of flavonoids in *C. asiatica* might be responsible for the suppression of RAAS activation. In addition, the high antioxidant property of *C. asiatica* showed a significant role in reducing oxidative stress in cardiomyocytes (Zainol et al., 2003; Pittella et al., 2009; Kumari et al., 2016). Ventricular hypertrophy and myocardial damage are among the complications of hypertension. In the presence of pressure overload due to systemic hypertension, the left ventricular wall thickens to minimize the wall stress (Drazner, 2011). Dysregulation of protein synthesis/processing within the endoplasmic reticulum during the hypertrophic response may be linked to the cardiomyocyte apoptosis process (González et al., 2018). Hence, the present study suggested that the protective effect of *C. asiatica* against hypertension was significant in the reduction in cardiac tissue damage in l-NAME treated group.

Our study also demonstrated the effects of captopril on SBP, serum NO, oxidative stress profile as well as a cardiac marker, and ACE activities. Captopril is one of anti-hypertensive drugs that belong to the ACE inhibitor group. This drug was chosen as a positive control since ACE inhibitors are the first line of treatment in hypertension and cardiac failure (Kementerian Kesihatan Malaysia, 2019) and it also possesses antioxidant activity (Bartosz et al., 1997; Benzie and Tomlinson, 1998). In the current study, captopril prevented a reduction in serum NO level, as well as an increase in SBP, cardiac and aortic TBARS content, serum BNP level, and cardiac ACE activities in the l-NAME-induced hypertensive rat model. The effects of captopril were comparable to that of *C. asiatica*. The results were supported by other studies showing that daily administration of captopril (5 mg/kg) could reduce hypertension and cardiac remodeling in rats with long-term exposure to l-NAME via inhibition on ACE (Maneesai et al., 2016b; Bunbupha et al., 2019). The mechanism of l-NAME leads to raising the blood pressure by inhibiting the eNOS activity, thus diminishes NO production. Hence, a drug like sodium nitroprusside that promotes NO release could be a more suitable choice as the positive control. However, it must be administered parenterally, making its daily administration a difficult routine (Hottinger et al., 2014).

We acknowledged other limitations in the current study. We only used a single dose of ethanolic extract of *C. asiatica*, guided from the results of our pilot study using three different doses; 300, 500 and 1,000 mg/kg (Supplementary Material). At the dose of 300 mg/kg, *C. asiatica* was reported to have high anti-oxidant properties (Muchtaromah et al., 2016) and at the dose of 500 mg/kg, this extract possessed significant diuretic property (Roopesh et al., 2011) which had the potential as an anti-hypertensive agent. Meanwhile, *C. asiatica* at the dose of 1,000 mg/kg exhibited a significant reduction in the ischemic area of cardiac tissues in rat model with post-myocardial infarction event (Pragada et al., 2004). Our pilot study showed that 500 and 1,000 mg/kg of ethanolic extract of *C. asiatica* significantly prevented the systolic blood pressure in l-NAME-induced hypertensive rats after 3 weeks of treatment and the lower dose was chosen for our main study. The rationale of using only a single dose of the extract is to minimize the number of animals needed in animal experimentation to comply with 3Rs (refinement, reduction and replacement) for best practice using animals. Future studies should be considered to establish a detailed dose-response relationship and to investigate different routes/timing of administration of the extract. Another limitation in this study is the assessment of vascular responsiveness, serial echocardiography, and histomorphology for cardiac and aortic tissues were not performed. The vascular reactivity test and serial echocardiography demonstrate the vascular and cardiac function changes. In contrast, a histomorphology test is a useful tool for assessing tissue remodeling development in hypertension and cardiac failure. Hence, additional studies are required to obtain these results. Nevertheless, this is the first study that investigated the role of *C. asiatica* in preventing hypertension and cardiac remodeling in the chronic NO deficient state. The present findings proposed that the amelioration of oxidative status and RAAS activity are the most likely mechanisms contributing to these protective effects. Given the protective potentials of this herbal plant, *C. asiatica* is highly advocated as a promising preventive approach in hypertension and its cardiac complication. Further studies are recommended to investigate the effect of *C. asiatica* in other hypertensive rat models or more severe stages of hypertension.

## Data Availability

The datasets presented in this study can be found in online repositories. The names of the repository/repositories and accession number(s) can be found in the article/Supplementary Material.
