# Supplementary figures and images for: Centella asiatica (L.) Urb. Prevents Hypertension and Protects the Heart in Chronic Nitric Oxide Deficiency Rat Model
Source: Front Pharmacol. 2021 Dec 3;12:742562. doi: 10.3389/fphar.2021.742562 (PMC8678489; doi:10.3389/fphar.2021.742562)

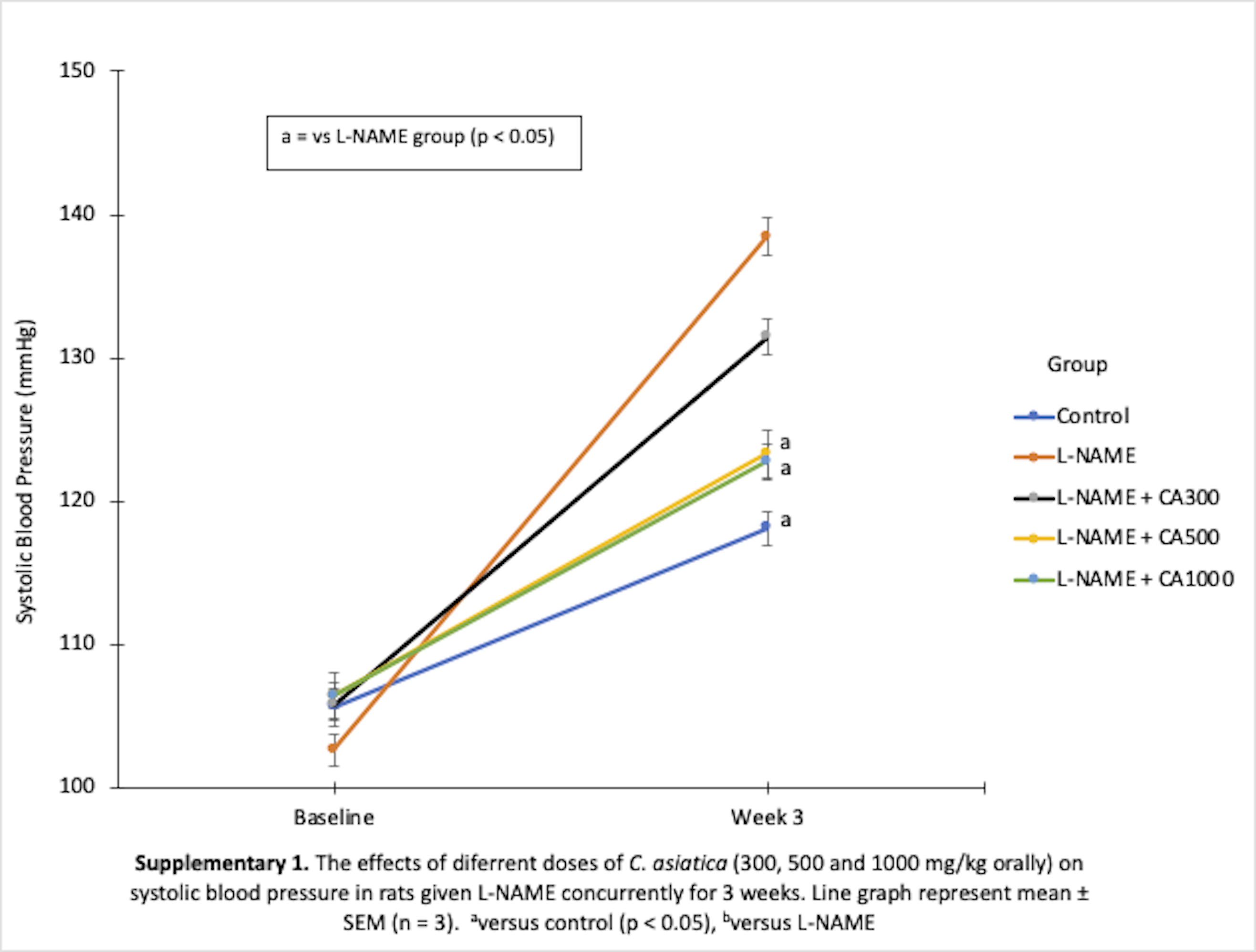

Supplement: Supplementary file 1 [file Image1.TIFF]
